# Transcranial focused ultrasound selectively increases perfusion and modulates functional connectivity of deep brain regions in humans

**DOI:** 10.3389/fncir.2023.1120410

**Published:** 2023-04-05

**Authors:** Taylor Kuhn, Norman M. Spivak, Bianca H. Dang, Sergio Becerra, Sabrina E. Halavi, Natalie Rotstein, Benjamin M. Rosenberg, Sonja Hiller, Andrew Swenson, Luka Cvijanovic, Nolan Dang, Michael Sun, David Kronemyer, Rustin Berlow, Malina R. Revett, Nanthia Suthana, Martin M. Monti, Susan Bookheimer

**Affiliations:** ^1^Department of Psychiatry and Biobehavioral Sciences, University of California, Los Angeles, Los Angeles, CA, United States; ^2^Department of Neurosurgery, University of California, Los Angeles, Los Angeles, CA, United States; ^3^UCLA-Caltech Medical Scientist Training Program, Los Angeles, CA, United States; ^4^Department of Psychology, University of California, Los Angeles, Los Angeles, CA, United States; ^5^Neuroscience Interdepartmental Program, University of California, Los Angeles, Los Angeles, CA, United States; ^6^Department of Psychological and Brain Sciences, Dartmouth College, Hanover, NH, United States; ^7^American Brain Stimulation Clinic, Del Mar, CA, United States; ^8^Department of Bioengineering, University of California, Los Angeles, Los Angeles, CA, United States

**Keywords:** transcranial focused ultrasound, functional connectivity, brain perfusion, amygdala, entorhina cortex

## Abstract

**Background:**

Low intensity, transcranial focused ultrasound (tFUS) is a re-emerging brain stimulation technique with the unique capability of reaching deep brain structures non-invasively.

**Objective/Hypothesis:**

We sought to demonstrate that tFUS can selectively and accurately target and modulate deep brain structures in humans important for emotional functioning as well as learning and memory. We hypothesized that tFUS would result in significant longitudinal changes in perfusion in the targeted brain region as well as selective modulation of BOLD activity and BOLD-based functional connectivity of the target region.

**Methods:**

In this study, we collected MRI before, simultaneously during, and after tFUS of two deep brain structures on different days in sixteen healthy adults each serving as their own control. Using longitudinal arterial spin labeling (ASL) MRI and simultaneous blood oxygen level dependent (BOLD) functional MRI, we found changes in cerebral perfusion, regional brain activity and functional connectivity specific to the targeted regions of the amygdala and entorhinal cortex (ErC).

**Results:**

tFUS selectively increased perfusion in the targeted brain region and not in the contralateral homolog or either bilateral control region. Additionally, tFUS directly affected BOLD activity in a target specific fashion without engaging auditory cortex in any analysis. Finally, tFUS resulted in selective modulation of the targeted functional network connectivity.

**Conclusion:**

We demonstrate that tFUS can selectively modulate perfusion, neural activity and connectivity in deep brain structures and connected networks. Lack of auditory cortex findings suggests that the mechanism of tFUS action is not due to auditory or acoustic startle response but rather a direct neuromodulatory process. Our findings suggest that tFUS has the potential for future application as a novel therapy in a wide range of neurological and psychiatric disorders associated with subcortical pathology.

## Introduction

Non-invasive methods for treating psychiatric and neurological disorders by modulating neural activity in humans include techniques such as transcranial magnetic stimulation (TMS) (Barker et al., 1985) and transcranial direct current stimulation (tDCS) (Perlson, 1945). However, these techniques are limited by their inability to target deep brain regions (e.g., amygdala or hippocampus (HC)), which are currently only effectively modulated by invasive, higher-risk deep brain stimulation (DBS) (Obeso et al., 2001). Here, we provide initial evidence that low intensity transcranial focused ultrasound (tFUS) can non-invasively modulate neural activity in the human amygdala and hippocampus by measuring changes in cerebral blood perfusion using arterial spin labeling (ASL) magnetic resonance imaging (MRI) and functional connectivity (FC) during simultaneous tFUS and blood oxygenation level dependent (BOLD) functional MRI (fMRI).

Focused ultrasound has recently been explored as a novel neuromodulation technology (Pasquinelli et al., 2019; Toccaceli et al., 2019; Stern et al., 2021). At high intensities, ultrasound can be used to cause ablations (e.g., for neurosurgical pallidotomy) (Moosa et al., 2019). Low intensity tFUS can penetrate the skull and dura (Hynynen and Jolesz, 1998), thereby affecting neuron populations in the brain, likely through cellular modulation (Tufail et al., 2011). By changing the parameters of the ultrasound such as pulse repetition frequency and duty cycle, it is possible to create potentiating or disruptive effects at the network level (Spivak et al., 2022), without also causing tissue damage via the heating effects seen at higher intensities (Schafer et al., 2021). Consequently, tFUS can circumvent the limitations of current neuromodulation techniques while maintaining high safety levels (Pasquinelli et al., 2019).

tFUS is thought to modulate neural activity either via mechanical stretching or neuronal intramembrane cavitation excitation (NICE). The mechanical stretching model suggests that tFUS physically stretches the cell soma resulting in membrane depolarization by way of voltage gated ion channel influx (Morris and Juranka, 2007; Tyler et al., 2008; Kubanek et al., 2016). Many ion channels have been shown to be influenced by ultrasonic stimulation, including mechanosensitive two-pore-domain potassium channels (Kubanek et al., 2016), and channels not typically classified as mechanosensitive (i.e., sodium and calcium voltage-gated channels) (Morris and Juranka, 2007). Alternatively, the NICE model proposes that tFUS causes spaces to form and disappear between the hydrophobic tails of the phospholipids comprising the cell bilayer (Plaksin et al., 2016). The law of conservation of charge establishes that greater distance between the charges (inside and outside the cell) results in greater electric potential, thereby changing the membrane potential. Whether this change is potentiating (akin to tDCS) or directly modulating (akin to TMS) is as of yet unclear.

Regardless of its precise mechanism, early histology and animal studies of tFUS demonstrate reversible physiologic effects on neuron clusters such as dentate gyrus and CA1 subregions (Tyler et al., 2008). Such effects include increased neuronal and astrocytic ionic conductance resulting in amplified cellular and synaptic activity, as measured by whole-cell patch clamps and confocal imaging of *ex vivo* neural cell cultures subjected to low intensity ultrasound (Tyler et al., 2008). Using real-time fMRI of macaque monkeys, tFUS produced transient disruption FC between the amygdala and its functional network (Folloni et al., 2019). In humans, tFUS has been shown to modulate BOLD signal in sensorimotor cortex (Ai et al., 2016), primary motor cortex (Ai et al., 2016, 2018), primary visual cortex (Lee et al., 2016) as well as the right inferior frontal gyrus and its functional network (Sanguinetti et al., 2020). Recently, tFUS in humans has been shown to modulate BOLD signal in basal ganglia regions including the globus pallidus (Cain et al., 2021b) and caudate (Ai et al., 2016), as well as the thalamus (Li et al., 2021) with associated changes in subjective reporting of pain and level of consciousness. When targeting a different thalamic nucleus, tFUS also appears to affect levels of consciousness in coma patients (Monti et al., 2016; Cain et al., 2021a). Importantly, nearly all of these studies compared BOLD signal before and after tFUS rather than reporting real-time changes in network connectivity during tFUS sonication. Neuroimaging data acquired simultaneously with tFUS administration is critical to maximize the likelihood of precise targeting of brain regions and engagement of their functional networks (Spivak and Kuhn, 2019). To our knowledge, no other studies report effects of tFUS on perfusion, BOLD or FC of the amygdala or entorhinal cortex (ErC) in humans. The capacity to successfully and non-invasively target and modulate these deep brain regions has wide-ranging implications for clinical neuromodulation of disorders involving anxiety, emotion regulation, learning and memory.

Non-invasive TMS has a large clinical effect size for Major Depressive Disorder (MDD), Posttraumatic Stress Disorder (PTSD), Obsessive-Compulsive Disorder (OCD) and Generalized Anxiety Disorder (GAD) (Cirillo et al., 2019). TMS, however, is unable to directly target deep brain structures such as the amygdala, which prior studies suggest is a meaningful target for treating anxiety disorders. Rather, TMS and tDCS indirectly engage deep brain regions via downstream modulation achieved by targeting dorsal cortical regions that are functionally connected to those deep targets. DBS of the amygdala has been shown to reduce hypervigilance in rodent models of PTSD (Langevin et al., 2010; Stidd et al., 2013). Additionally, in a single human patient with PTSD, DBS of the amygdala resulted in increased pleasant memories and regulated sleep (Langevin et al., 2016). The demonstrated potential of DBS to modulate amygdalar activity and associated behavior provides promise for similar findings in non-invasive tFUS.

Similarly, DBS of the ErC improved memory in a small sample of patients with Alzheimer’s disease (AD) (Suthana et al., 2012; Suthana and Fried, 2014). In rodent models, direct stimulation of the perforant pathway—the afferent connections arising from the ErC projecting to the dentate gyrus and CA1 region of the HC—enhanced memory and HC neurogenesis (Toda et al., 2008). Similar findings have been reported using theta burst electrical stimulation in the ErC and perforant pathway area prior to learning, which improved subsequent memory (Titiz et al., 2017). Replicating these ErC DBS findings using tFUS would be a major advancement in non-invasive brain stimulation therapies for amnestic syndromes such as amnestic mild cognitive impairment (aMCI) and AD.

In the present study, we administered tFUS simultaneously with BOLD fMRI to examine brain circuit modulation in two key deep brain structures: the amygdala, implicated in many anxiety and mood disorders (Fox and Shackman, 2019), and the ErC, implicated in memory formation (Montchal et al., 2019) and impaired episodic memory in AD (Gómez-Isla et al., 1996; Olajide et al., 2021). Similar to the macaque study (Folloni et al., 2019) and directly related to our goal of disrupting amygdala connectivity, we used a sonication paradigm intended to inhibit/disrupt (Spivak et al., 2022) amygdala activity. Conversely, we also used a sonication paradigm hypothesized to excite/stimulate (Spivak et al., 2022) the ErC with the goal of increasing ErC connectivity. In order to ensure accurate targeting and engagement of the target brain region, we used longitudinal arterial spin labeling (ASL) MRI to quantify tFUS-related perfusion changes in the targeted brain regions.

### Hypotheses

**FIGURE 1:**
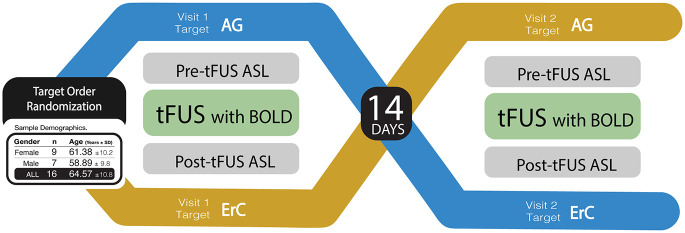
Study design. A visual representation of the randomized, double-blind, within-subject crossover study design. Participants completed two study visits, separated by a 14-day between-session window. During each session, participants underwent a baseline MRI assessment of regional blood flow using Arterial Spin Labeling (ASL) MRI. Thereafter, participants received tFUS in the MRI with simultaneously collected blood oxygenation level-dependent (BOLD) MRI. After tFUS was administered, ASL was collected again to compare to baseline. The brain region targeted during each study session was randomized and counterbalanced across participants such that 47% received amygdala tFUS during the first study session and 53% received ErC tFUS during their first session. Examples of amygdala and ErC tFUS targeting are provided, as well as a chart detailing sample demographics.

This study sought to demonstrate proof-of-principle evidence that tFUS can evidence target-specific brain perfusion and functional connectivity changes indicative of neuromodulation. We were able to perform perfusion MRI before and after tFUS. Because tFUS is MRI compatible, we were also able to collect resting state FC MRI simultaneously during tFUS administration. We performed tFUS on a group of sixteen healthy aging adults targeting both regions (amygdala and ErC) two weeks apart, order counterbalanced and data collected and examined blindly, to determine the effect on perfusion, regional brain activity and associated FC (Figure 1). We expected that we would see tFUS-induced 1) increased perfusion only in the targeted brain region and not the control region 2) changes in brain function measured using BOLD associated with tFUS and 3) modulation of FC of the targeted region and its functionally connected regions.

## Results

### tFUS-related increased regional perfusion

**FIGURE 2:**
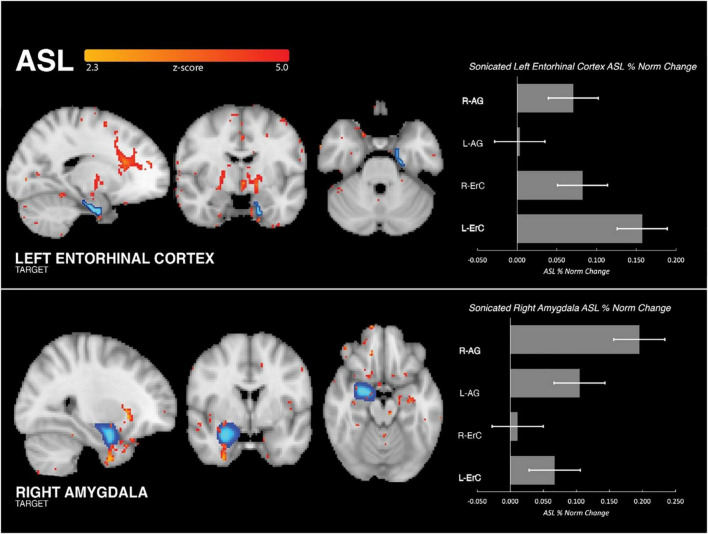
Group perfusion findings. **(Left Column)** Analysis of ASL MRI demonstrated that tFUS was associated with significant increase in perfusion to the targeted region and not the control region (i.e., when targeting ErC, increased perfusion to ErC and not amygdala, and vice versa). Increased perfusion was also seen in functionally connected regions. For ErC: anterior cingulate cortex, medial prefrontal cortex and basal ganglia regions including anterior thalamus. For amygdala: medial prefrontal cortex and ventral forebrain. **(Right Column)** Bar graph illustrating the mean, normalized percent perfusion change associated with tFUS in the four regions of interest: right amygdala, left amygdala, right entorhinal cortex, left entorhinal cortex. When sonicating the left ErC, increased perfusion was found in the sonicated left ErC and not the right ErC or bilateral amygdala. Similarly, when sonicating the right amygdala increased perfusion was found in the sonicated right amygdala and not the left amygdala or bilateral ErC.

Within-subject, partial volume corrected ASL MRI demonstrated increased perfusion in the region of the brain targeted by tFUS as compared to the control region. When sonicating the right amygdala, increased perfusion was found in the sonicated amygdala (Cohen’s d: 0.97, mean ASL signal change = 19.52%; SD_ROI_ = 0.93, SD_sample_ = 0.38, p < 0.001; Figure 2) and not in the left ErC (Cohen’s d: 0.2; mean ASL signal change = 6.72%; SD_ROI_ = 0.34, SD_sample_ = 0.29, p > 0.1) or right ErC (Cohen’s d: 0.11; mean ASL signal change = 1.13%; SD_ROI_ = 0.33, SD_sample_ = 0.26, p > 0.1). Similarly, when sonicating the left ErC, increased left ErC perfusion (Cohen’s d: 0.8; mean ASL signal change = 15.75%; SD_ROI_ = 1.02, SD_sample_ = 0.23, p < 0.001; Figure 2) without increased right amygdala (Cohen’s d: 0.08; mean ASL signal change = 7.07%; SD_ROI_ = 0.35, SD_sample_ = 0.25, p > 0.1) or left amygdala perfusion (Cohen’s d: 0.05; mean ASL signal change = 3.35%; SD_ROI_ = 0.35, SD_sample_ = 0.17, p > 0.1) was found. Additionally, significantly increased perfusion was found only in the targeted brain structure ipsilateral to the tFUS transducer and not in the contralateral homologue region. Specifically, the sonicated right amygdala evidenced increased perfusion following sonication (as described above) while the left amygdala, which was not targeted, did not display a significant change in perfusion (Cohen’s d: 0.31; mean ASL signal change = 10.48%; SD_ROI_ = 0.72, SD_sample_ = 0.23, p > 0.1). Similarly, only the targeted left ErC, and not the right ErC, evidenced statistically significant tFUS-related increased perfusion (Cohen’s d: 0.1; mean ASL signal change = 8.24%; SD_ROI_ = 0.77, SD_sample_ = 0.36, p > 0.1). Further, statistically significantly increased (all p’s < 0.001) perfusion was observed in regions known to be functionally connected to the targeted areas. Amygdala-focused tFUS increased amygdala and medial prefrontal cortex (PFC) perfusion (Cohen’s d: 0.32); mean ASL signal change = 11.63%). ErC-focused tFUS increased ErC perfusion as well as perfusion in the HC (Cohen’s d: 0.17; mean ASL signal change = 6.20%), anterior cingulate (Cohen’s d: 0.26; mean ASL signal change = 14.42%), and bilateral basal ganglia regions including the thalamus (Cohen’s d: 0.26; mean ASL signal change = 14.38%).

### tFUS-related brain activation

**FIGURE 3:**
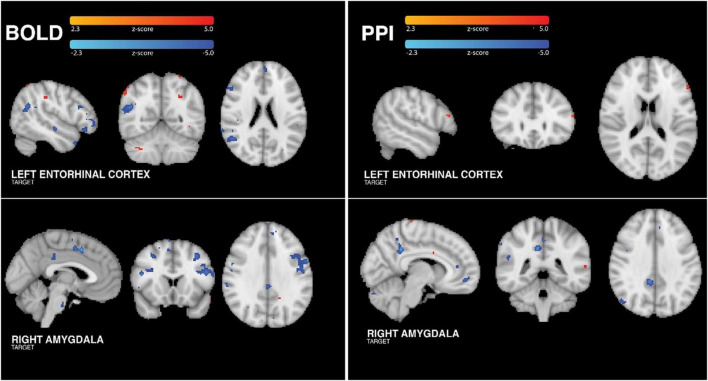
Group BOLD findings. **(Left Column)** Analysis of tFUS on BOLD showed significantly increased activity in small areas of the temporo-occipito-parietal junction, occipital cortex and right cerebellum. Additionally, left ErC tFUS was associated with significantly reduced BOLD in anterior frontal, anterior temporal, and posterior parietal cortices. tFUS targeting the right amygdala resulted in significantly decreased BOLD activity in the posterior cingulate, pre-sensorimotor cortex, dorsolateral prefrontal cortex and pons. **(Right Column)** When sonicating the ErC, group PPI analysis revealed tFUS-related increased connectivity between the left ErC and left dorsolateral prefrontal cortex. Group PPI analysis of data collected when sonicating the amygdala revealed tFUS-related decreased FC between the right amygdala and posterior cingulate, anterior cingulate, medial prefrontal and posterior parietal regions. PPI control analyses confirmed that these findings were specific to the target region.

Functional BOLD MRI data was collected at the same time as tFUS sonication. The main effect of tFUS on brain activity was modeled using a traditional block-design paradigm matching the alternating 30s blocks during which the transducer was on and off. The results of this analysis (Figure 3) revealed that tFUS targeting the right amygdala resulted in significantly decreased BOLD activity in the amygdala, posterior cingulate, supplemental motor area cortex, dorsolateral prefrontal cortex and pons. There were no areas of significantly increased BOLD when targeting the right amygdala (all FDR-corrected *p*-values > 0.05).

When targeting the left ErC, tFUS was associated with significantly increased BOLD in small areas of the ErC, temporo-occipito-parietal junction, occipital cortex and right cerebellum. Additionally, left ErC tFUS was associated with significantly reduced BOLD in anterior frontal, anterior temporal including entorhinal/parahippocampal areas, and posterior parietal cortices. Importantly, neither amygdala or ErC analysis revealed significant or trending towards significant changes in BOLD in either auditory cortex (all FDR-corrected p values > 0.05), suggesting that these effects were indeed due to a neuromodulatory effect of tFUS and not due to an auditory startle response (as shown by Guo et al. (2018) and Sato et al. (2018)) or an acoustic reaction to bone conduction from the vibration of the transducer (Dobrev et al., 2017). For both targets, all participants demonstrated positive correlations between their unique individual BOLD changes and the group changes, which is to say that all participants demonstrated BOLD changes in the expected direction consistently with the group findings.

### tFUS-related network connectivity changes

For each participant, a standard MNI152 atlas mask of the targeted brain region was used as the seed region for a psychophysiological interaction analysis (PPI), which shows regions of modulated connectivity between brain areas during sonication but not during rest. PPI analyses were conducted for each perfusion seed (amygdala and ErC) on the BOLD data collected simultaneously with the tFUS sonication experiment. By analyzing the PPI of both sonication target seeds, regional specificity of FC changes was assessed. As such, one seed corresponded to the brain region targeted by tFUS during the BOLD acquisition, and the other seed corresponded to the brain region targeted during the other tFUS session (control region). As a second control analysis, we also ran PPI analyses using the target seed on the BOLD data collected when targeting the control region.

Sonicating the right amygdala, group PPI analysis (Figure 3) revealed tFUS-related decreased FC between the right amygdala and posterior cingulate, anterior cingulate, medial prefrontal and posterior parietal regions. Using the ErC as a control region, we confirmed that these tFUS-evoked FC changes were specific to the right amygdala. Importantly, PPI analysis of the BOLD data collected when targeting the amygdala using an ErC seed did not yield any significant FC changes associated with the ErC (all p’s > 0.05 FDR corrected). Similarly, PPI analyses conducted using the BOLD acquired during ErC tFUS with the amygdala seed did not yield any amygdala-related FC changes (all FDR-corrected *p*-values > 0.05).

Group PPI analysis of data collected when sonicating the left ErC revealed tFUS-related increased connectivity between the left ErC and left dorsolateral prefrontal cortex (Figure 3). Again importantly, PPI analyses using the above amygdala seed applied to the BOLD data collected while the ErC was sonicated did not yield any amygdala-related FC changes (all p’s > 0.05 FDR corrected). Finally, PPI analyses conducted using the BOLD acquired during amygdala tFUS with the ErC seed did not yield any ErC-related FC changes (all FDR-corrected *p*-values > 0.05).

Across all PPI analyses, there was no significant effect found in the auditory cortex (all FDR-corrected *p*-values > 0.05). For both targets, all participants demonstrated positive correlations between their unique individual PPI-based functional connectivity changes and the group changes, which is to say that all participants demonstrated connectivity changes in the expected direction consistently with the group findings.

## Discussion

In these investigations we combined tFUS with ASL MRI and simultaneous BOLD fMRI to examine the impact of focused ultrasound on deep brain areas of the human brain. The results indicate differential impacts of tFUS on amygdala and ErC perfusion, BOLD activity and FC. The findings suggest that tFUS can preferentially increase regional blood flow and modulate network connectivity of subcortical regions, potentially in a desired direction. tFUS sonication parameters hypothesized to disrupt activity yielded decreased FC in the amygdala network including prefrontal cortex, cingulate and brainstem. Conversely, tFUS sonication parameters hypothesized to increase functional activity resulted in generally, though not exclusively, increased BOLD activity and FC in the targeted ErC and its network (e.g. HC). tFUS of the ErC also activated visual regions, likely through the integration of visual input through the ErC via efferent downstream projections (Schultz et al., 2015). Interestingly, both tFUS protocols resulted in increased perfusion in the targeted brain region - not the control region nor the contralateral homolog of the targeted region. This double dissociation in perfusion and FC supports our hypothesis that the modulatory effects of tFUS are focal and directly related to the targeted region. Supporting the conclusion that these findings were due to neuromodulatory effects of tFUS, none of our analyses found engagement of the auditory cortex. Indeed, no participant reported any auditory or visual effects during sonication. Without evidence of auditory cortex activation associated with tFUS, it is unlikely that our findings are related to auditory startle response (38) or acoustic reactions to bone conduction (39). Effective targeting of tFUS is critical for successful modulation of the desired brain region(s) and associated cognitive and/or affective functions and ASL. fMRI appears to be an effective way to measure target engagement in the brain. The present findings suggest that ASL is an effective method of confirming engagement of tFUS-targeted brain regions.

This study also supports prior ones which found that tFUS can be used to modulate BOLD activity in cortical and subcortical regions (Ai et al., 2016, 2018; Lee et al., 2016; Monti et al., 2016; Sanguinetti et al., 2020; Cain et al., 2021a,b; Li et al., 2021), adding the amygdala and entorhinal cortex to the list of deep brain regions that can be modulated with tFUS. However, our findings were not directionally consistent with those of Cain et al., who sonicated the thalamus using the 100Hz paradigm (ErC) and the 10 Hz paradigm (amygdala). Cain et al. found reduced perfusion and FC using the same sonication parameters as we used when targeting the ErC (100Hz) and no FC changes using the parameters we used when targeting the amygdala (10Hz). These different findings are possibly due to the differences in vasculature (e.g., thalamostriate vs. major/middle cerebral arteries) and connectivity of the thalamus compared to the medial temporal lobe structures in the present study. Further, our findings contribute to this literature by providing additional evidence that tFUS selectively increases regional perfusion while modulating both regional activity and functional connectivity. These results extend the possible clinical applications of tFUS by confirming the ability of tFUS to engage deep brain regions in humans important for emotion regulation as well as memory formation and retrieval. Our amygdala sonication results closely align with those of the amygdala tFUS study conducted in macaques (Folloni et al., 2019), which was influential to the design of this project. Interestingly, those investigators found that the tFUS-related FC changes persisted for the 80-minute duration of their resting state fMRI (rs-fMRI) assessment. It will be important to determine the duration of tFUS effects in humans for the design of clinical trials and eventual clinical implementation of tFUS. Additional work in a larger sample with varying time windows between tFUS application and post-tFUS evaluation also is required.

The differential effects of amygdala tFUS present an opportunity for further investigation of non-invasive techniques for the treatment of anxiety disorders and other psychiatric pathologies. For example, it seems likely that the timing of amygdala tFUS will be relative to the acquisition, generalization and extinction of fear and anxiety. If tFUS can disrupt the new learning of fear, then it may be useful in emergency departments to help lessen the severity of or prevent the development of PTSD. Alternatively, if tFUS is able to accelerate the extinction of previously learned fear, then it could be an adjunctive treatment for patients with already established anxiety-related syndromes such as GAD, PTSD, Social Anxiety Disorder (SAD), panic disorder, or OCD. tFUS may offer a treatment for “treatment-resistant” anxiety and mood disorders. Further work in this area will be important to determine the extent to which tFUS is a viable “affective neural prosthetic” for interventional use in psychiatric disorders.

Similarly, tFUS of the ErC could be a meaningful non-invasive intervention for diseases affecting learning and memory, such as MCI and AD. As with the amygdala, further research is needed to determine the optimal timing for ErC sonication. One study in humans found improvement in spatial memory performance when DBS to the entorhinal cortex was administered during learning trials (Suthana et al., 2012). It’s possible that tFUS may have an enhanced effect on learning and subsequent memory when sonication is applied concurrently during the initial learning and/or retrieval of memory events. The sonication parameters used in this study hypothesized to excite/engage the ErC network resulted in both increased and decreased BOLD and functional connectivity. Further exploration of ErC tFUS sonication parameters and sonication-to-stimuli timing protocol, as well as ErC tFUS effects in participants with learning and memory disorders, is needed to validate and optimize tFUS as a non-invasive cognitive prosthetic tool.

No adverse events occurred during this study. Participants were followed every day for three days following each tFUS session and exhibited no negative reactions, including physical discomfort or heightened anxiety. Given its nascency in humans, it will be important to monitor these and other pertinent safety variables. A recently published review of the current findings on tFUS safety in humans and animals reported that adverse events following tFUS are rare, occurring only in studies that administered tFUS at intensities above the currently approved limit for use in humans (Pasquinelli et al., 2019). With FDA approval and oversight, one group recently administered tFUS to temporal lobe in patients with intractable temporal lobe epilepsy scheduled for surgical resection of the epileptogenic tissue of the temporal lobe (Stern et al., 2021). Histological examination following excision of the previously sonicated tissue did not indicate any tissue damage, including thermal or cavitation effects, following tFUS intensities up to eight times higher than that used in this study. Another study sonicating prepared slices of brain tissue saw no damage until intensities nearly 20x the intensities used in present study (Spivak et al., 2021). These outcomes support a conclusion that low intensity tFUS does not work via a thermal or tissue damaging cavitation mechanism, and is safe for use in humans. Additional safety work is needed to further establish safety guidelines for clinical use across a variety of patient populations, brain targets and clinical use cases.

The preliminary nature of this study entails limitations shared by all early-stage studies of novel technology. One limitation is that the somewhat small sample size leads to decreased statistical power. However, the moderate to large effect sizes of the tFUS-related brain changes indicate that the study was sufficiently powered to detect changes in perfusion, BOLD activity and functional connectivity, at both the group and single-participant level. Further, the sample comprised only individuals undergoing healthy aging. It did not compare tFUS effects between healthy individuals and those with neurologic and psychiatric disorders associated with the targeted brain regions (e.g., GAD, PTSD, and AD). Additionally, during the simultaneous tFUS-BOLD analysis, an assumption underlying the statistical PPI model was that tFUS resulted in instantaneous effects on the BOLD signal which were appropriately modeled using a block design which followed the on-off blocks of the sonication administration. It is possible that effect of tFUS builds up over time and therefore an evolving matrix, rather than an on-off block, model would be more appropriate. However, to our knowledge, this is not yet known in the field and our attempts generate, using our data, an empirical model of tFUS effects other than this block design were unsuccessful. Therefore, currently, the block design appeared to be the best model. However, larger-scale future analyses should further investigate this possibility. Similarly, longitudinal BOLD analyses are warranted to help determine the time scale of the tFUS effects on functional connectivity. The macaque study that informed the amygdala portion of the present study demonstrated ongoing FC effects of tFUS throughout the 2 hour duration of the BOLD study.

The purposes of this study were to demonstrate the feasibility of an alternative to other more invasive and less effective treatments for neurologic and psychiatric disorders affecting learning, memory, anxiety and emotion regulation, and to suggest directions for further research. Other research has shown that tFUS can reliably target desired areas of the deep brain without engaging nearby structures. For example, the ErC and amygdala lie within 1cm of one another in the brain, yet neither was affected by targeting the other. That being so, it is unclear whether all participants were stimulated in the exact same sub-region within the amygdala and ErC. Even though perfusion data suggested that the majority of the targeted brain region was sonicated, given that sub-regional activation may vary subject-to-subject, further work is necessary to enhance targeting precision and more fully understand the impact of tFUS on regional subnuclei. In this vein, the amygdala is a larger target region than the ErC and therefore the sonication likely effected a different proportion of target tissue. Advances in focal beam technology and the impact of varying sonication shapes and sizes will likely assist in the effective clinical implementation of tFUS technology.

## Materials and methods

### Study design

Participants completed two experimental sessions conducted exactly two weeks apart. Pre-tFUS, simultaneous-tFUS, and post-tFUS MRI data were collected during each experimental session. tFUS was performed in the MRI scanner and targeted one brain region per experimental session: one session targeting the right amygdala and one targeting the left ErC (Figure 1). The order of the brain regions targeted by tFUS was randomized and counterbalanced across participants. Participants were blinded to which brain region was sonicated during each session; they were only aware that the tFUS transducer was placed on the left side of their head during one session (ErC) and the right side of their head during the other session (amygdala). Study staff performing statistical analyses were blinded to the tFUS target associated with the data. For MRI data, this involved masking the data so that the tFUS transducer could not be seen on the images prior to beginning the processing pipeline. As such, the design was a double-blind randomized, within-subjects crossover clinical trial. Prior to participant enrollment and data collection, both studies were registered in the National Clinical Trials archive (ClinicalTrials.gov, 2018a,b).

### Demographics

From twenty-one screened adults, eighteen healthy adults were recruited for this study. Due to motion artifacts rendering some MRI data unusable, two participants were removed from analyses, yielding a final sample size of sixteen healthy aging adults. These participants were, on average, 61.38 (7.75) years old, 56% female, and were 37% Caucasian American, 31% Latinx American, 19% African American and 13% Asian American. Given our group’s plan to expand the ErC tFUS protocol into studies involving participant populations with neurodegenerative diseases, healthy aging adults were specifically recruited for this project.

### Screening procedures

All procedures were in accordance with the Declaration of Helsinki and approved by the University of California, Los Angeles (UCLA) Institutional Review Board prior to enrollment. All participants provided written informed consent. Screening protocols were adapted from the study Mapping the Human Connectome During Typical Aging (Bookheimer et al., 2019) to obtain a sample representative of healthy adult aging. Included in this screening was a set of questions ensuring safety to undergo MRI examination. Potential participants were excluded for prior diagnosis and/or treatment of major psychiatric disorders (e.g., schizophrenia, bipolar disorder), neurological disorders (e.g., stroke, brain tumors, Parkinson’s Disease), or severe depression that required treatment for 12 months or longer in the past five years. In individuals 60 years and older, potential participants were excluded based on impaired cognitive abilities as assessed by a cognitive screener: the Telephone Interview for Cognitive Status modified (TICS-M) (Knopman et al., 2010). To be eligible for the study, potential participants were required to score 30 or greater on the TICS-M, after adjusting for educational background. After obtaining written informed consent, the Montreal Cognitive Assessment (Nasreddine et al., 2005) was administered to ensure that participants who did not earn the minimum score for their age bracket were excluded from the study.

### MRI-guided tFUS targeting

**FIGURE 4:**
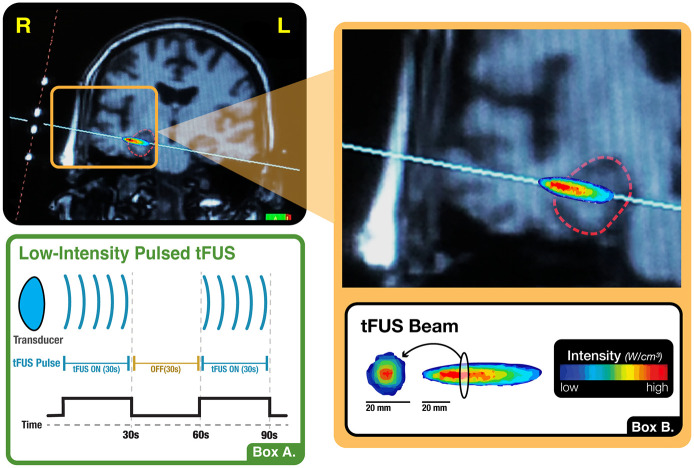
tFUS paradigm. **(A)** Illustration of the present study’s sonication block design, wherein the transducer alternated between 30-s blocks of active stimulation and no stimulation. This cycle occurred ten times, totaling five minutes of non-consecutive tFUS. **(B)** Visualization of the shape, orientation and intensity distribution of the tFUS beam. When measured in a water tank using a hydrophone, the tFUS beam appears ellipsoid in shape with a central focus of higher intensity and a surround of lower intensity. The longitudinal dimension is approximately 2 cm in length while the cross section, which also evidences a higher intensity centroid, is approximately 0.5 cm in diameter.

The tFUS sonications were delivered using a single-element transducer placed above the ear at the temporal window, one of the thinnest parts of the skull bone, and targeted using real-time structural MRI navigation inside the MRI. tFUS of the amygdala used sonication parameters hypothesized to decrease or disrupt activity in the sonicated emotion region. This was modeled in part off of the Foloni study in Macaques (Folloni et al., 2019) and was done based on the hypothesis that disruption of amygdala and its functional network may serve as the foundation for investigating tFUS clinical applications in anxiety disorders. tFUS of the ErC used sonication parameters hypothesized to increase activity in the targeted memory region. This was based on the hypothesis, based in part on our collaborators work in ErC DBS (Suthana et al., 2012; Suthana and Fried, 2014), that stimulation of the ErC may lead to improved learning and memory. Both paradigms used a 5% duty cycle, in 10 cycles of 30 s on, 30 s off, for a total of 5 min of non-consecutive tFUS (Figure 4). The paradigm targeting the amygdala used a 5 ms pulse width repeated at a 10 Hz pulse repetition frequency (PRF), while the paradigm targeting the ErC used a 0.5 ms pulse width repeated at a 100 Hz PRF. In both instances, the fundamental frequency was 0.65 MHz and the I_spta.3_ was 720 mW/cm^2^, which was determined by applying the derating equation with a derating factor of 0.3 dB/cm-MHz. Prior testing using cadaveric skulls in degassed water has shown that the skull acts to broaden the −6dB focal width by 1.5 mm and lengthen the −6dB axial focal length by 1.4 mm with a minimal lateral shift of less than 1mm (Schafer et al., 2021).

**FIGURE 5:**
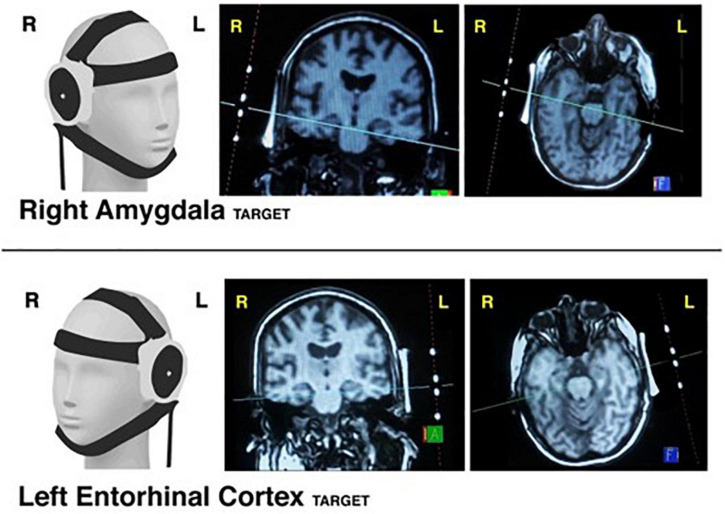
tFUS targeting. Visualization of transducer placement on 3D model (1^st^ column) when targeting the right amygdala **(Top Row)** and left entorhinal cortex **(Bottom Row)**. Examples of MRI-console guided targeting using transducer fiducial markers are provided in coronal view (2^nd^ column) and axial view (3^rd^ column).

tFUS was performed inside the MRI scanner using typical targeting approaches for low intensity tFUS (Spivak and Kuhn, 2019). This involved a 30-s SCOUT imaging sequence to visualize the tFUS transducer and its orthogonal line of targeting into the brain. The tFUS transducer had a focal sonication depth of 65 mm (BrainSonix Corp., Sherman Oaks, CA, USA (Schafer et al., 2021)). The MRI scanning console computer was used to visualize the transducer and, using fiducial markers built into the transducer, a line orthogonal to the center of the transducer was drawn on screen into the brain at 65 mm depth from the interface of the transducer and gel pad (Figure 5). The transducer was then manually moved as necessary to correct its position such that the targeted brain region was ultimately confirmed via SCOUT MRI as either the right amygdala or left ErC. Specifically, we attempted to target the centromedian aspect of the amygdala by aiming the targeting line through the middle of the body of the amygdala. We attempted to target the interface of the ErC and the perforant pathway by aiming the orthogonal line through the central axis of the angular bundle, which carries the perforant pathway, and subsequently the central region of the ErC.

### Neuroimaging acquisition

All MRI data were collected using a 3T Siemens MAGNETOM Prisma fit scanner (Siemens Medical Solution, Erlangen, Germany) located at the UCLA Center for Cognitive Neuroscience. ASL, multi-slice BOLD and T1 anatomical scans were collected from sixteen healthy aging adults. In order to accommodate the tFUS transducer into the MRI head coil, the 20-channel head coil was used for all acquisition sequences. This required minor modification to MP-RAGE scan borrowed from the Lifespan Human Connectome Project (Bookheimer et al., 2019) to ensure compatibility with the 20-channel coil (rather than 32-channel used in the HCP). ASL scans were collected before and after tFUS with a pulsed ASL sequence using 3.0 mm slices, FOV = 192 mm (AP) x 120 mm (FH), TR = 4,600 ms, TE = 16.18 ms, flip angle = 180^o^, bolus duration = 700 ms, inversion time = 1,990 ms, FAIR-QII Perfusion, 1 average, pre-scan normalization filter, gray, white and fat suppression filters and 1.5 mm x 1.5 mm x 3 mm voxels and total scan time of 4 min 59 s for each ASL sequence. Simultaneous tFUS BOLD data were collected using an optimized SMS GRE EPI sequence involving TR = 700 ms, TE = 33 ms, flip angle = 70^o^, FOV = 192 mm (AP) x 135 mm (FH) and 2.5 mm isotropic voxels with total scan time of 11 min 49 s. Framewise Integrated Real-time MRI Monitoring (FIRMM) (Dosenbach et al., 2017) was used during the collection of all BOLD data to monitor for participant motion. Prior to tFUS administration, structural MP-RAGE T1-weighted scans were acquired with 120 1.0-mm sagittal slices, FOV = 256 mm (AP) x 192 mm (FH), matrix = 256 × 192, TR = 450 ms, TE = 10 ms, flip angle = 8^o^, and voxel size = 1.0 mm x 0.94 mm x 0.94 mm. All images were quality controlled and visually inspected prior to being preprocessed and analyzed. The tFUS transducer was placed inside the MRI head coil for the resting state fMRI scan during which the tFUS was administered. For all other MRI sequences, the tFUS transducer was removed from the head coil and the scanner. It took approximately five minutes to extract the participant from the scanner, either place or remove the transducer, and replace the participant in the scanner.

### Perfusion analysis

Pulsed Arterial Spin Labeling (PASL) scans produce a perfusion image with voxel values representing local perfusion rates. For each subject, pre-stimulation PASL images were linearly registered to each subject’s T1. Perfusion images were processed by using the BASIL (Chappell et al., 2008) toolbox, includimmg partial volume correction, then transferred to MNI space using non-linear registration in FSL. Using FSL Version 6.0,^1^ a voxel-wise comparison of pre-vs-post tFUS sonication was conducted individually for each subject by subtracting the registered pre-sonication perfusion map from the post-sonication perfusion map. A 2 × 2 repeated measures analysis of variance (ANOVA), corrected for multiple comparisons using False Discovery Rate (FDR), compared the longitudinal perfusion changes between amygdala sonication and ErC sonication at the voxel-wise level. Results of this ANOVA are reported along with the mean and standard deviation perfusion change within each region of interest as well as across the study sample.

### Simultaneous tFUS-BOLD analysis

10-minute tFUS experiments were administered as BOLD data was collected inside the MRI scanner. Sonication-synced BOLD functional data processing included motion correction to the mean image, spatial smoothing (Gaussian Kernel FWHM = 5 mm), high-pass temporal filtering (*t* > 0.01 Hz) and regression-based removal of outliers (ICA-Aroma (Pruim et al., 2015)). To examine the main effect of tFUS on BOLD, a whole brain general linear model was set up specifying the onset and duration (30s) of the tFUS sonication blocks. Resulting statistical maps estimating the voxel-wise magnitude of neural activation associated with tFUS were corrected for multiple comparisons using FDR and thresholded at z = 2.3 and FDR-correct *p* < 0.001.

Further, to examine tFUS-related network connectivity, a seed-based approach was used to examine whole brain connectivity with the tFUS target of interest and compare between stimulation (on-off) conditions using PPI modeling. FSL FEAT module was used to conduct these analyses. The seed regions used for this PPI analysis were right amygdala, automatically segmented from each participant’s structural MRI using FSL First (Patenaude et al., 2011), and left ErC, adopted from a standard, functionally-derived atlas (Maass et al., 2015). Both ROIs were registered initially to each participant’s fMRI. Group analyses were then conducted following registration of the functional data to standard MNI space. The mean time series from this seed region in the preprocessed BOLD image was extracted using fslmaths and entered as the first explanatory variable. The block-design entered as the second explanatory variable was computed from the timed on-off tFUS sonication blocks (main effect of tFUS described above). The third explanatory variable was the interaction of the tFUS-target-seed mean time series and the sonication on-off blocks. The time series variable was centered at zero and the block design was centered at the mean. The resulting statistical maps were corrected for multiple comparisons using FDR and thresholded at z = 2.3 and FDR-corrected *p* < 0.001. We also ran correlation analyses to determine the number of participants whose individual tFUS-related changes were associated with the group findings.

## Data availability statement

The raw data supporting the conclusions of this article will be made available by the authors, without undue reservation.

## Ethics statement

The studies involving human participants were reviewed and approved by UCLA IRB. The patients/participants provided their written informed consent to participate in this study.

## Author contributions

TK, SBo, MM, NSu, NSp, and RB: conceptualization. TK, SBe, SBo, MM, NSu, RB, NSp, BD, and SHi: methodology. TK, SBe, NSp, BD, SHi, SBo, BR, AS, LC, MS, DK, and MR: investigation. TK, BD, SBe, SHa, and NR: visualization. TK, SBo, and MR: funding acquisition. TK, SBo, BD, and NSp: project administration. TK, SBo, NSp, BD, and MR: supervision. TK, SBe, NSp, BD, SBo, SHa, and NR: writing – original draft. All authors: writing – review and editing.

## Footnotes

1
www.fmrib.ox.ac.uk/fsl
